# Dietary Polyphenols in Prevention and Treatment of Prostate Cancer

**DOI:** 10.3390/ijms16023350

**Published:** 2015-02-03

**Authors:** Rahul K. Lall, Deeba N. Syed, Vaqar M. Adhami, Mohammad Imran Khan, Hasan Mukhtar

**Affiliations:** 1 Department of Food Science, University of Wisconsin, Madison, WI 53706, USA; E-Mail: rklall@wisc.edu; 2 Department of Dermatology, School of Medicine and Public Health, University of Wisconsin, Madison, WI 53706, USA; E-Mails: dsyed@dermatology.wisc.edu (D.N.S.); vadhami@dermatology.wisc.edu (V.M.A.); ikhan@dermatology.wisc.edu (M.I.K.)

**Keywords:** dietary polyphenols, prostate cancer, chemoprevention, phenolic acid, flavonoids, stilbenes, curcuminoids

## Abstract

Prostate cancer is the most prevalent disease affecting males in many Western countries, with an estimated 29,480 deaths in 2014 in the US alone. Incidence rates for prostate cancer deaths have been decreasing since the early 1990s in men of all races/ethnicities, though they remain about 60% higher in African Americans than in any other group. The relationship between dietary polyphenols and the prevention of prostate cancer has been examined previously. Although results are sometimes inconsistent and variable, there is a general agreement that polyphenols hold great promise for the future management of prostate cancer. Various dietary components, including polyphenols, have been shown to possess anti-cancer properties. Generally considered as non-toxic, dietary polyphenols act as key modulators of signaling pathways and are therefore considered ideal chemopreventive agents. Besides possessing various anti-tumor properties, dietary polyphenols also contribute to epigenetic changes associated with the fate of cancer cells and have emerged as potential drugs for therapeutic intervention. Polyphenols have also been shown to affect post-translational modifications and microRNA expressions. This article provides a systematic review of the health benefits of selected dietary polyphenols in prostate cancer, especially focusing on the subclasses of polyphenols, which have a great effect on disease prevention and treatment.

## 1. Introduction

Prostate cancer (PCa) is the most prevalent cancer in the male population in Western countries. Based on recent evidence, it is the second leading cause of cancer-related death among men in the US [1]. Age, family history, genetic factors, lifestyle, environmental influences, and diet are some of the most important risk factors associated with PCa. Rising incidence rates of PCa have been observed over the last few decades, largely due to screening and early detection procedures [2]. Recently, diet-derived polyphenols have received tremendous attention among nutritionists, food scientists, and consumers for their health-promoting effects, including their use in the chemoprevention of PCa [3]. The health effects of dietary polyphenols depend directly on the amount consumed and on their bioavailability, which is influenced by chemical structure (polymerization, esterification, acetylation, methylation, and esterification), food matrix, and excretion back into the intestinal lumen. Furthermore, neither the absorption efficacies of all polyphenols are the same nor are their effects on the various signaling pathways that they modulate. The absorption efficacy is dependent on the hepatic enzymes and the composition of the intestinal microflora within the human body [4,5,6]. Polyphenols have also been reported to modulate key proteins in the signaling cascades related to differentiation, proliferations, metastasis and apoptosis [7,8].

**Figure 1:**
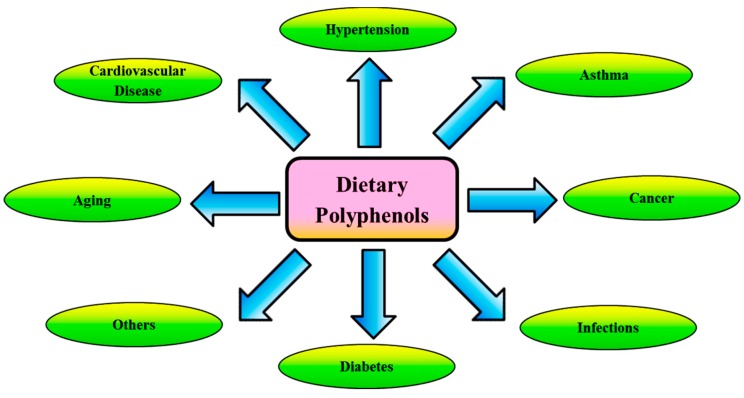
Beneficial health effects of dietary polyphenols. Polyphenols have been widely explored and are potent antioxidants. Polyphenols neutralize the destructive reactivity of undesired reactive oxygen species (ROS)/reactive nitrogen species (RNS) produced during the metabolic processes in the human body.

Dietary polyphenols are naturally occurring food compounds found in fruits, vegetables, cereals and beverages. To date, more than 8000 compounds have been identified in the human diet based on their chemical structures [9]. These molecules are identified as the secondary metabolites of plants that contain one or more hydroxyl (–OH) groups attached to -ortho, -meta or -para positions on a benzene ring. These metabolites are generally involved in defense against ultraviolet radiation, the effects of various environmental pollutants, and hostility from pathogens [10]. The long-term consumption of a polyphenol-rich diet has shown promise against cardiovascular diseases (CVDs), neurodegenerative diseases, diabetes, cancer, and many others in epidemiological studies as shown in Figure 1. This review focuses on the current understanding of the biological effects of selected dietary polyphenols, which are being reported as instrumental in their effect on treatment and prevention of PCa.

The following key words were used in the initial search strategy using PubMed: polyphenols, diet, natural products, PCa, tumor and chemoprevention; the search was augmented by a profound exploration of polyphenols involved in the treatment and prevention of PCa.

## 2. General Structure and Classes of Dietary Polyphenols

**Figure 2:**
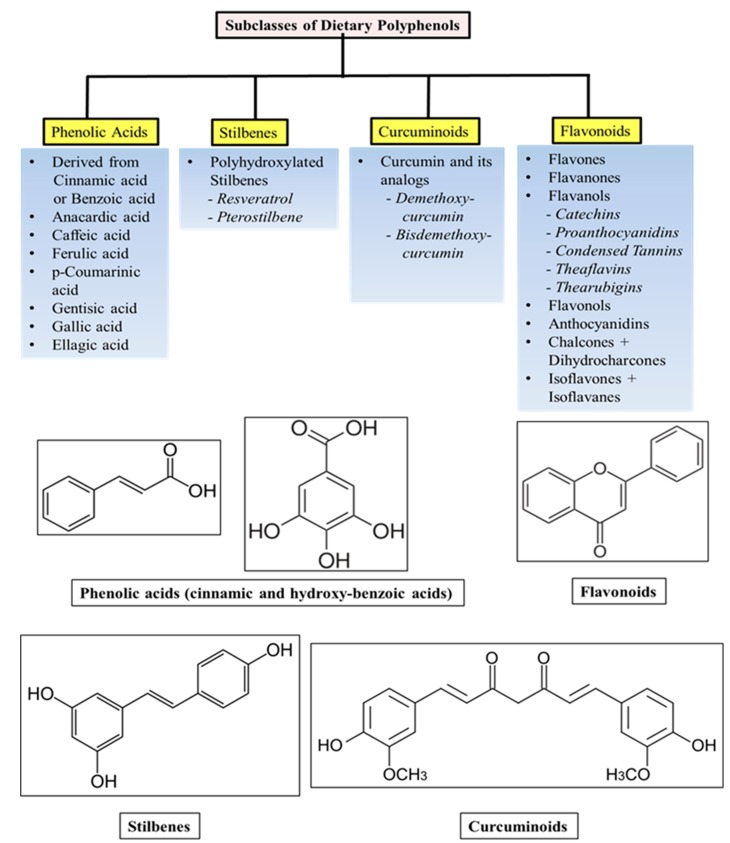
Subclasses and general structures of dietary polyphenols.

Polyphenols are polyhydroxylated phytochemicals and share common chemical structures such as conjugated closed rings and hydroxyl groups [11]. Most abundant polyphenols found in diets may be classified into various groups as a function of their chemical structure and orientation of the number of phenol rings bound to one another. They are subdivided into four main subclasses: phenolic acids, stilbenes, curcuminoids and flavonoids, of which phenolic acids and flavonoids account for 30% and 60% respectively [12,13]. The different subclasses and general chemical structures of the polyphenols are illustrated in Figure 2.

Phenolic acids are further categorized into hydroxy-benzoic and hydroxy-cinnamic acids. Phenolic acids account for about a third of the polyphenolic compounds in our diet and are found in all plant material, but are particularly abundant in acidic-tasting fruits. Caffeic acid, gallic acid and ferulic acid are some common phenolic acids.

Flavonoids are the most abundant polyphenols in human diet and share a common basic structure consisting of two aromatic rings, which are bound together by three carbon atoms that form an oxygenated heterocycle. Biogenetically, one ring usually arises from a molecule of resorcinol, and the other ring is derived from the shikimate pathway [12,13].

Stilbenes contain two phenyl moieties connected by a two-carbon methylene bridge. Most stilbenes in plants act as anti-fungal phytoalexins, compounds that are synthesized only in response to infection or injury. The most extensively studied stilbene is resveratrol, which we have discussed below.

**Figure 3:**
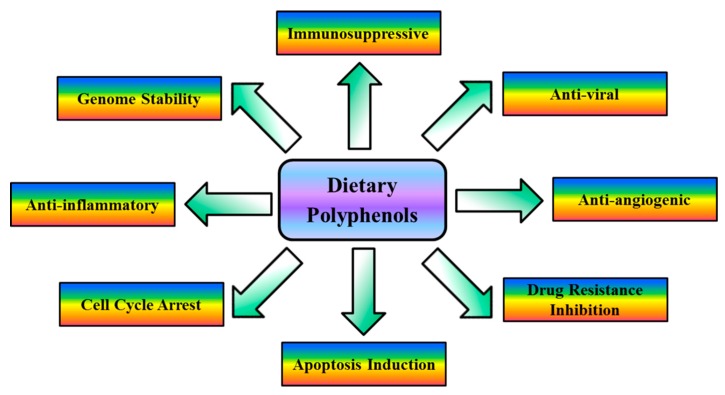
Cellular, molecular, and biochemical actions of dietary polyphenols. Dietary polyphenols target signaling molecules, including growth factors, transcription factors, cytokines, enzymes, and genes regulating apoptosis. Dietary polyphenols play an important role in inflammation, apoptosis, angiogenesis and auto-immune diseases.

Curcumin and various analogs of curcumin contain the linear diarylheptanoid curcuminoid. These compounds are natural phenols and produce a pronounced yellow color. The different chemical groups increase the solubility of curcuminoids, making them more suitable for drug formulation. The known cellular, molecular, and biochemical actions of dietary polyphenols have been summarized in Figure 3.

## 3. Dietary Polyphenols in Prostate Cancer

This section provides an overview of selected dietary polyphenols (based on their subclasses) which have been used in studies directed towards PCa prevention and treatment.

### 3.1. Phenolic Acids

Phenolic acids are composed of hydroxy-cinnamic and hydroxy-benzoic acids and account for 30% of dietary polyphenols [7]. They are ubiquitous to plant material and sometimes present as esters and glycosides. They have anti-oxidant activity as chelators and free radical scavengers with special impact over hydroxyl (–OH) and peroxyl radicals, superoxide anions, and peroxynitrites. Gallic acid, one of the most studied and promising compounds in PCa research, belongs to the hydroxy-benzoic group. Gallic acid is the precursor of many plant-derived tannins, while cinnamic acid is the precursor of hydroxy-cinnamic acids [14,15].

#### 3.1.1. Anacardic Acid

Anacardic acid (AA; 6-pentadecylsalicylic acid) is the active phenolic lipid found in the *Amphipterygium adstringens* plant. It possesses anti-inflammatory, anti-cancer, anti-oxidative and anti-microbial functions. The bark of this plant is widely used in traditional medicines for treatment of gastric ulcers, gastritis and stomach cancers [16]. In PCa, AA is reported as a natural inhibitor of non-specific histone acetyltransferase and has been shown to inhibit prostate tumor angiogenesis by targeting the proto-oncogene tyrosine-protein kinase (Src)/focal adhesion kinase (FAK)/rhodopsin (Rho) guanosine triphosphate (GTP)ase signaling pathway [17]. AA affects multiple steps of tumor angiogenesis including endothelial cell viability, migration, adhesion, and differentiation both* in vitro* and* in vivo*. The AA-mediated effect and mechanism on PCa cells is based on its ability to inhibit cell proliferation and induce G1/S cell cycle arrest and apoptosis. AA inhibits androgen receptors (AR), activates tumor suppressor protein p53 and cyclin-dependent kinase (CDK) inhibitor-1/p21, and regulates the transcription of other related target genes [18]. 

#### 3.1.2. Caffeic Acid

Caffeic acid (CA; 3,4-dihydroxycinnamic) is one of the hydroxy-cinnamate metabolites universally present in plant tissues. CA is found in many food sources, including coffee drinks, blueberries, apples and cider. Besides acting as a cancer inhibitor [19,20], it also possesses anti-oxidant and anti-bacterial activities* in vitro* and can contribute to the prevention of atherosclerosis and other CVDs [21]. CA has been reported to inhibit AR signaling and subsequent inhibition of cell proliferation of human androgen-dependent PCa cells.

Some derivatives of CA have also shown potent cytotoxic and anti-proliferative effects and dihydrotestosterone (DHT)-stimulated prostate specific antigen (PSA) secretion [22]. CA-phenyl ester (CAPE) enhances anti-proliferative and cytotoxic effects of docetaxel (DOC) and paclitaxel (PTX) in PCa cells attributed to CAPE augmentation of DOC and PTX proapoptotic effects in addition to CAPE-induced alterations in estrogen receptors (ER)-α and ER-β abundance [23,24]. CAPE significantly reduced protein kinase-B/Akt, extracellular signal-regulated kinases (ERK), and ER-α phosphorylation. CAPE-mediated inhibition of Akt phosphorylation was more prominent in cells expressing ER-α such as PC3 compared to LNCaP. CAPE suppressed the proliferation of LNCaP, DU145, and PC3 human PCa cells in a dose-dependent manner.

Overexpression of Akt1 and c-Myc significantly blocked the antiproliferative effects of CAPE. CAPE administration may be useful as an adjuvant therapy for cancers that are driven by the p70S6K and Akt signaling networks [25]. CAPE, a known inhibitor of NFκB can inhibit interleukin (IL)-6 secretion induced by tumor necrosis factor (TNF)-alpha, thereby suppressing signal transducers and activators of transcription (STAT)-3 translocation [26]. CAPE treatment suppressed proliferation, colony formation, and cell cycle progression in PC3 cells. CAPE decreased protein expression of cyclin D1, cyclin E, SKP2, c-Myc, Akt1, Akt2, Akt3, total Akt, mammalian target of rapamycin (mTOR), B-cell lymphoma (Bcl)-2, retinoblastoma protein (Rb), as well as phosphorylation of Rb, ERK1/2, Akt, mTOR, glycogen synthase kinase (GSK)3α, GSK3β, and PDK1, but increased protein expression of KLF6 and p21^Cip1^ in PC3 cells [27]. Taken together, evidence shows that CA has multiple protective effects, which can be further explored and developed towards PCa chemoprevention.

#### 3.1.3. Ellagic Acid

Ellagic acid (EA; 4,4',5,5',6,6'-Hexahydroxydiphenic acid) is a polyphenolic compound present in fruits and berries such as pomegranates, strawberries, raspberries, and blackberries. It has anti-carcinogenic, anti-oxidant and anti-fibrosis properties. It is responsible for more than 50% of the anti-oxidant activity of pomegranate juice and for the beneficial effects of EA in PCa [28,29,30,31]. EA treatment of LNCaP cells induced a significant decrease in heme oxygenase (HO)-1 and -2, cytochrome P450 (CYP) 2J2 expression, and vascular endothelial growth factor (VEGF) and osteoprotegrin (OPG) levels. Similarly, CYP4F2 and CYPA22 were significantly downregulated by EA treatment, suggesting that EA interfered with multiple biological processes involved in angiogenesis and metastasis in PCa cells [32].

Recently, apoptotic pathways involved in EA-mediated chemoprevention were reported. Apoptosis was induced by downregulation of anti-apoptotic proteins, SIRT1, HuR, and HO-1. EA modulated apoptosis inducing factor (AIF), resulting in an increase in ROS levels and caspase (CASP)-3, while reducing transforming tumor growth factor (TGF)-β and IL-6 [33]. EA reduced proliferation by inhibiting mTOR and decreasing levels of β-catenin. EA slightly decreased matrix metalloproteinase (MMP)-2 but had no effect on MMP-9 in PC3 cells. Non-toxic concentration of EA was shown to inhibit invasion and motility of PCa cells through its action on protease activity [34]. Treatments with EA induced differentiation by causing significant reduction in chromogranin-A, p-Rb, DNMT-1, and p-Akt levels, along with increased p75 neurotrophin receptor expression. EA also induced DNA damage in PCa cells in a dose-dependent manner [35]. Pomegranate juice (PJ) containing EA, along with other components, has been shown to inhibit PCa metastasis.

Two initial exploratory clinical studies investigating proprietary pomegranate products reported a trend of effectiveness in increasing PSA doubling time in patients with PCa [36,37]; however, another clinical study did not support these results [38]. Recently, a group evaluated the PJ blends to investigate the contrasting clinical evidence between these two studies. Their results showed that daily doses of PJ in the latter study contained very little concentrations of gallic acid and punicalagin compared to the concentrations found in the earlier two studies. The authors confirmed that not just pomegranate but the amount of co-active compounds in the PJ blend along with EA was responsible for its clinical effectiveness [39].

#### 3.1.4. Gallic Acid

Gallic acid (GA; 3,4,5-trihydroxybenzoic acid) is ubiquitously present either in free form or, more commonly, as a constituent of tannins, namely gallotannins [40]. Some of the natural products found in nature that are rich in GA are strawberries, pineapples, bananas, lemons, red and white wines, gallnuts, sumac, witch hazel, tea leaves, oak bark and apple peels [41]. Biologically, GA possesses anti-bacterial, anti-viral, anti-inflammatory, and anti-oxidant properties [41,42,43,44]; anti-melanogenic activity is also present via the inhibition of tyrosinase activity [45]. Anti-cancer activity of GA has been reported in leukemia, oral tumor and esophageal cancer cells [46,47]. GA inhibited cell viability in DU145 and 22Rν1 PCa cells in a dose-dependent manner via induction of apoptosis [48].

Regarding GA’s ability against PCa, studies have shown both anti-cancer and cancer chemopreventive effects in human PCa DU145 cells* in vitro* and the transgenic adenocarcinoma of the mouse prostate (TRAMP) model, respectively [49,50]. GA inhibited the tumor growth in DU145 and 22Rν1 PCa xenografts in nude mice and decreased microvessel density, as compared to controls in both models [51]. Penta-O-galloyl-beta-d-glucose (5GG), which consists of a glucose molecule on which five –OH groups are esterified with GA, has been shown to suppress tumor growth via inhibition of angiogenesis [52] and STAT-3 activity in PCa cells [53]. 5GG arrested cells at the G1 phase, induced apoptosis, inhibited lipopolysaccharide-induced NFκB activation, fatty acid synthase (FAS) expression and suppressed cell invasion by reducing MMP-9 expression [54].

Mechanistic studies of 5GG-mediated regulation of MMP-9 showed activation of EGF-induced c-jun *N*-terminal kinase and subsequent suppression of NFκB nuclear translocation. 5GG also reduced epidermal growth factor receptor (EGFR) expression through the proteasome pathway and suppressed invasion and tumorigenesis in nude mice implanted with PC3 cells [55]. 5GG’s role as a novel inhibitor of DNA polymerases was studied and the results showed that 5GG induced PCa S-phase arrest through DNA replicative blockage and induced G1 arrest via cyclin D1 downregulation [56]. Another analog of GA, theaflavin-3-3'-digallate (TF3), and 5GG together showed inhibition of rat liver microsomal 5alpha-reductase activity, which catalyzes the conversion of testosterone to a more active androgen, DHT which then subsequently binds to AR and functions inside the nucleus to regulate specific gene expression. Furthermore, TF3 and 5GG reduced androgen-responsive LNCaP cell growth, inhibited expression of AR, and lowered androgen-induced PSA and FAS protein levels.

### 3.2. Stilbenes

Stilbenes or stilbenoids are a well-known class of naturally occurring polyphenols. Stilbenes are chemically characterized by their core structure of 1,2-diphenylethylene. Most stilbenes are stress metabolites produced in plants and act as anti-fungal phytoalexins, compounds that are only synthesized in response to an infection or injury. These plant defense compounds have tremendous potential in biological and cellular processes applicable to human health [57]. Stilbenes are reported to be potentially important cancer chemoprotective agents, being able to inhibit cellular events associated with carcinogenesis, including tumor initiation, promotion, and progression [58].

#### 3.2.1. Piceatannol

Piceatannol (PT; trans-3,4,3',5'-tetrahydroxystilbene) is a naturally occurring polyphenol present in rhubarb, berries, peanuts, sugar cane, wine and grape skins. PT, a metabolite biotransformed from resveratrol (RSV), has been demonstrated to exert anti-inflammatory, anti-carcinogenic and cardioprotective effects [59]. *In silico* and biochemical analyses have identified quinone reducatase 2 (QR2) as a target of PT. PT-mediated inhibition of cell proliferation and induction of apoptosis was comparable to RSV. PT interacted with QR2 at the same site as RSV, forming an H-bond with asparagine-161. The anti-cancer effect of PT observed in PCa cells was shown to be QR2-dependent, as PT-mediated inhibition of proliferation and QR2 activity were much lower in QR2-knockdown cells relative to QR2 expressing cells. The study suggested PCa prevention by RSV to be partially attributed to its conversion to PT [60].

PT inhibits the migration/invasion of DU145 PCa cells possibly mediated by decrease in IL-6/STAT-3 signaling [61]. PT delayed G1 cell cycle progression of DU145 cells via the inhibition of CDK2 and CDK4 [62]. PT was found to induce apoptosis in DU145 human PCa cells via activation of extrinsic death receptors and intrinsic mitochondrial-dependent pathways [63]. Recently a study showed that inhibition of MMP-9 by PT decreased the invasive potential of DU145 cells. PT inhibits TNF-α-induced invasion by suppression of MMP-9 activation via Akt-mediated NFκB pathways in DU145 PCa cells [64]. Another study showed* in vivo* evidence that PT, when administered orally, inhibits tumor formation, growth, and diminished cell colonization in LNCaP PCa xenografts [65]. PT has been shown to suppress the activation of some transcription factors including NFκB, which plays a central role as a transcriptional regulator in response to cellular stress caused by free radicals, ultraviolet radiation, cytokines, or microbial antigens.

PT inhibits Janus kinase-1 (Jnk-1), a key member of the STAT pathway crucial in controlling cellular activities in response to extracellular cytokines, and is involved in inflammation and carcinogenesis. The anti-tumor, anti-oxidant, anti-inflammatory, and pharmacological properties of PT suggests that PT might be a potential biomolecule for PCa prevention; however, more data are needed on its bioavailability and toxicity in humans [66].

#### 3.2.2. Pterostilbene

Pterostilbene (PTER; trans-3,5-dimethoxy-4-hydroxystilbene), an anti-oxidant found mainly in berries and grapes, has gained much attention due to its chemopreventive and potential therapeutic effects reported in a variety of cancer types [67]. PTER-isothiocyanate, a conjugate of PTER inhibits the AR-regulated pathways in PCa cells. The conjugate significantly repressed cell proliferation, induced apoptosis by modulating phosphoinositide 3-kinase (PI3K)/Akt and mitogen-activated protein kinase (MAPK)/ERK pathways, arrested cell cycles, abrogated DHT induced activation, and down regulated AR expression in LNCaP cells [68]. PTER treatment inhibited cell proliferation in a dose-dependent manner in p53 wild type LNCaP and p53 null PC3 cells. PTER activated adenosine monophosphate-activated protein kinase (AMPK) in both p53 positive and negative human PCa cells, resulting in a decrease in activity and expression of lipogenic enzymes FASN and acetyl-CoA carboxylase (ACC). PTER increased the expression level of p53 and subsequently enhanced the expression level of p21, resulting in cell-cycle arrest in LNCaP cells. It is proposed that induction of p21 promoted growth arrest and exerted a protective affect after AMPK activation [69].

PTER induced apoptosis, cell cycle, and PSA in the human androgen-responsive LNCaP cells [70]. The effects of PTER against PCa were also studied in highly metastatic androgen-independent LNCaP cells and showed that PTER is an effective inducer of apoptosis based on flow cytometry and microscopic analysis of cell surface morphology. The authors investigated PTER’s effect upon three specific markers of mitochondrial apoptosis—Bcl-2, BAX and CASP-3—and found that pterostilbene decreased Bcl-2 expression by 2- to 2.5-fold and increased expression of BAX and CASP-3 by 2- and 3-fold, respectively [70]. This study reported PTER inhibits cell viability in LNCaP cells and causes cell cycle arrest at the G1/S-phase after 72 h of treatment. Furthermore, the anti-carcinogenic effects of PTER were seen upon two CDK inhibitors, CDNK1A and CDNK1B, which are essential to G1/S-phase regulation. PTER was found to up-regulate both CDNK1A and CDNK1B at a concentration of 25 μM in LNCaP cells. PTER treatment inhibited elevated PSA mRNA expression in LNCaP cells with a minimal concentration of 1 μM [70]. Further, PTER treatment inhibited elevated PSA levels that were hormonally induced groups, DHT and 17β-estradiol. PTER decreased Akt activation, MMP expression, and further contributed to anti-carcinogenesis. Akt and MMP are both associated with cancer cell proliferation and metastasis and down-regulated expression of the cancer marker α-methylacyl-CoA racemase.

A recent study demonstrated that dietary stilbenes are effective regulators of metastasis-associated protein (MTA1)/nucleosome, remodeling deacetylase-mediated p53 acetylation, apoptosis, and angiogenesis in PCa xenografts [71]. MTA1 has the additional advantage of being sensitive to pharmacologically safe dietary compounds. On the basis of strong* in vitro* and* in vivo* evidence, the authors proposed PTER to be explored as a lead compound for potent target-specific treatment of MTA1-overexpressing advanced PCa. PTER increased glutathione (GSH) peroxidase, GSH reductase and total GSH by 1.4-, 1.6-, and 2.1-fold, respectively. Furthermore, PTER increased levels of ROS by 5-fold and nitric oxide production by 6-fold. These findings indicated that PTER modified the anti-oxidant profile of PCa cells, leading to a cellular environment that is conducive to apoptosis [72]. Based on these cumulative findings, PTER possesses potent effects in both hormonal-responsive and hormonal-independent PCa* in vitro* and* in vivo*, suggesting its chemotherapeutic implications in PCa.

#### 3.2.3. Resveratrol

Resveratrol (RSV; 3,4',5-trihydroxystilbene), one of the best studied stilbenes, is found largely in grapes, blueberries, peanuts, pistachios and hops. A product of grapes, red wine also contains significant amounts of RSV [10,73]. RSV exists both in *cis*- and *trans*-stereoisomeric forms of which the *trans*-isomer is biologically active [74,75]. RSV induces a broad range of effects on cell phenotypes. Ample evidence on RSV indicates inhibition of cancer cell growth, induction of cell cycle arrest, and apoptosis in various PCa cell lines [76,77,78]. RSV is known to induce differentiation in certain cell types [79,80,81].

COX-2 catalyzes the conversion of free arachidonic acid to prostaglandins, which can stimulate cell proliferation, promote angiogenesis, and suppress apoptosis, all of which promote malignancy [82,83,84]. RSV expresses anti-inflammatory activity by directly inhibiting COX-2 activity and suppressing NFκB by up-regulating MAPK-phosphatase-5 [85,86]. RSV has also been reported to reduce expression of MMPs, which are responsible for tumor invasion and metastasis and also decreases the levels of VEGF, resulting in angiogenesis inhibition [87,88,89,90,91]. RSV has the ability to increase sensitivity of PCa cells to ionizing radiation, which has potential, in combination with radiotherapy, for clinical applications [92,93,94,95,96,97].

Another recent report suggests Zn, in combination with RSV, as a novel approach for PCa management. Zn is abundantly available in healthy prostates, but with PCa progression, it reduces significantly [98]. RSV, in combination with Zn, was reported to increase the total cellular Zn and intracellular free labile Zn in normal human prostate epithelial cells [99]. In addition, an increase of Zn levels in plasma was reported in healthy adult rats administered with RSV. These studies suggest that RSV may influence Zn homeostasis, possibly via enhancing intracellular Zn accumulation [100]. The anti-cancer potential of RSV has been summarized in many* in vitro* and* in vivo* studies previously published [101]. RSV is well tolerated, but an optimal dose has not yet been determined. Another study recently confirmed that even though RSV has shown anti-cancer potential in various experimental studies reported to date, there is so far no concrete evidence to support the use of the compound for PCa treatments outside of clinical trials. The main reason for this caveat is that there is not enough clinical evidence to justify a recommendation for the prophylactic administration of RSV [102].

### 3.3. Stilbenes

Curcuminoids, curcumin, and their structurally related compounds are comprised of phenolic yellowish crystalline powder and are used to provide flavor and color to spice blends. Nutraceuticals (foods with medicinal potential) are prepared and consumed all across the world and are active in the prevention and treatment of various diseases including PCa [103]. Curcuminoids found in turmeric contain three principal components—curcumin, demethoxycurcumin and bisdemethoxycurcumin—of which curcumin is the most abundant and potent [104,105,106,107].

#### 3.3.1. Curcumin

Curcumin and its derivatives have been reported to possess anti-inflammatory, anti-oxidative and anti-carcinogenic properties [108]. Curcumin was shown to inhibit proliferation in both androgen-dependent and androgen-independent PCa cell lines [109]. Curcumin inhibited several cell signaling pathways including NFκB, TNFR pathways. Curcumin and its derivatives demonstrated anti-cancer properties by inhibiting enzymes like COX-2, MMPs, mTOR, protein kinase C, and EGFR [110,111,112,113,114]. Curcumin inhibits PCa cell viability and induces cell apoptosis. The authors report that curcumin downregulates the expression of the inhibitor of DNA binding (Id)-1 mRNA and protein in PC3 cells, a key signaling molecule in PCa carcinogenesis and metastatic progression [115]. Curcumin was shown to inhibit proliferation and migration of human PCa cells.

Curcumin significantly suppressed phosphorylation of ERK1/2 and VEGF expression modulating the osteopontin/integrin-αvβ3 signaling pathway. It also caused MMP-9 activation associated with angiogenesis via regulation of secretion of VEGF and angiostatin in PC3 cells [116]. Curcumin analogues have been reported to be more effective in inhibiting human PCa cells and to retard the growth of human PC3 xenografts in immuno-compromised mice, as compared to curcumin alone [117,118]. Curcumin as a modulator of ER activity is an effective agent and has demonstrated protection against PCa invasion and metastasis [119]. Several* in vitro* and* in vivo* studies have provided evidence regarding the efficacy of curcumin against PCa; however, further studies directed towards the development of curcumin analogues/nanoparticles are needed, through which bioavailability of curcumin may be enhanced for prevention or reducing the development of PCa [120,121].

#### 3.3.2. Demethoxycurcumin and Bisdemethoxycurcumin

Demethoxycurcumin (DMC) and bisdemethoxycurcumin (BDMC), analogs of curcumin, have been reported to modulate inflammatory signaling and cell proliferation to the same extent as curcumin. The relative potency for suppression of TNF-induced NFκB activation reported is curcumin > DMC > BDMC, suggesting the critical role of methoxy groups on the phenyl ring of curcumin. DMC and BDMC induced GSH to a similar extent as curcumin. Production of GSH correlates with suppression of NFκB activation and induction of cell proliferation through a ROS independent mechanism [122]. DMC has been reported to show the most efficient cytotoxic effects on PC3 cells. DMC activates AMPK and decreases activity of lipogenic enzymes FASN and ACC. DMC downregulates heat-shock protein (HSP)-70 and increases the activity of CASP-3. In addition, DMC treatment activates AMPK in PCa cells, which, in turn, regulated the HSP70/EGFR pathways. These findings demonstrate that AMPK pathways have a significant influence on DMC-mediated inhibition of tumor viability [123]. DMC inhibits migration of PC3 cells in both a dose- and time-dependent manner. DMC has also been reported to prevent against proliferation and apoptosis of PCa cells via CASP-3 routes. The activity of MMP-2 is suppressed, suggesting correlation between migration and invasion of PCa cells [124].

### 3.4. Flavonoids

Flavonoids comprise over 4000 varieties and account for about 60% of structurally-related dietary polyphenols, which are widely present in plants and ingested in varying degrees in the diet. Their chemical structure contains 2-benzene rings linked to three carbon atoms that form an oxygenated heterocycle [125]. Flavonoids are classified into flavonols, flavones, isoflavones, anthocyanidins, chalcones, and dihydrochalcones. The flavonols themselves are subdivided into cathechins, proanthocyanadins, theaflavins, and thearubigins [126]. Several beneficial properties have been attributed to these dietary compounds, including anti-oxidant, anti-inflammatory, and anti-carcinogenic effects. Flavonoids have shown potential to protect against viral infections, as well as several diseases such as diabetes, CVDs, inflammatory and neurological diseases [127,128].

#### 3.4.1. Apigenin

Apigenin (APG; 4',5,7,-trihydroxyflavone) is a naturally occurring plant flavone abundantly present in common fruits and vegetables such as grapefruits, plant-derived beverages, parsley, onions, chamomile, oranges, tea and wheat sprouts. The most common source of APG consumed as a single ingredient in herbal tea is chamomile, prepared from the dried flowers of *Matricaria chamomilla* [129,130]. Recently, APG has been increasingly recognized as a cancer chemopreventive agent. Numerous studies have explored the possible cancer chemopreventive effect of APG based on its potent anti-oxidant, anti-mutagenic, anti-inflammatory, anti-viral and purgative effects [131]. The promising role of APG was summarized in various cancers including PCa [132].

*In vitro* and* in vivo* studies indicate that APG mediated growth inhibitory responses are due to the inhibition of histone deacetylases (HDACs), specifically HDAC1 and HDAC3. This effect was observed both at the protein level as well as localized hyperacetylation of histone H3 on the p21 promoter, a condition that manifests HDAC-mediated therapeutic resistance [133]. APG induced up-regulation of p21, followed by subsequent inhibition of polo-like kinase (PLK)-1 transcription, resulting in apoptosis of PCa cells [134]. APG acts as an inhibitor of adenine nucleotide translocator (ANT)-2, an ADP/ATP translocator which up-regulates death receptors (DR)-5 at the post-transcriptional level and sensitizes malignant PCa tumor cells to apoptosis-inducing ligands (Apo2L)/TNF-related apoptosis-inducing ligands (TRAIL), whereas ANT-2 silencing leads to the enhancement of Apo2L/TRAIL mediated apoptosis [135]. APG treatment of androgen-refractory human PCa PC3 and DU145 cells resulted in dose-dependent reduction of X-linked inhibitor of apoptosis protein (X-IAP), c-IAP1, c-IAP2, and survivin protein levels. APG resulted in decreased cell viability and induction of apoptosis accompanied by decreases in Bcl-xL, Bcl-2 and increases in the active form of BAX proteins. APG resulted in inhibition of class 1 HDACs and HDAC1 protein expression, thereby increasing the acetylation of Ku70 and the dissociation of BAX resulting in apoptosis of PCa cells [136].

APG effectively suppresses PCa progression in spontaneous TRAMP mice by attenuating insulin-like growth factor (IGF)-1/IGF binding protein-3 signaling associated with inhibition of p-Akt and p-ERK1/2, resulting in inhibition of invasion and progression of PCa. APG showed marked inhibition of VEGF, urokinase-type plasminogen activator, MMP-2, and MMP-9, which coincided with tumor growth inhibition and complete absence of metastasis in TRAMP mice [137]. A study was performed to investigate inhibitory effect of APG on TGF-β-induced VEGF production. The authors reported that APG inhibited VEGF along with TGF-β1-induced phosphorylation via mothers against decapentaplegic homolog (Smad)-2/3 and Src/FAK/Akt pathways, providing insight into a novel molecular mechanism underlying the anti-angiogenic potential of APG [138]. Recently, APG has been reported to inhibit PCa progression in the TRAMP mouse model via targeting PI3K/Akt/forkhead box FoxO pathways. APG-treated mice showed decreased phosphorylation of Akt and FoxO3a, which correlated with reduced binding of FoxO3a. It also reduces proliferation by lowering Ki-67 and cyclin D1 along with an increase in FoxO responsive proteins like BIM and p27/Kip1 [139]. Oxidative stress is linked to a progression of PCa and human prostate is vulnerable to DNA damage due to oxidation. APG has been shown to preferentially accumulate in the nuclear matrix, binding particularly to the nucleic acid bases, and has the ability to reduce oxidative DNA damage in prostate epithelial cells [140].

#### 3.4.2. Epigallocathechin-3-gallate

Green tea is an aqueous mixture of dried unfermented leaves of *Camellia sinensis* and has shown to possess anti-mutagenic, anti-bacterial, hypocholesterolemic, anti-oxidant, anti-tumor and cancer preventive properties. Green tea is comprised of polyphenolic compounds like epigallocatechin-3-gallate (EGCG), epigallocatechin (EGC), epicatechin-3-gallate (ECG), and epicatechin (EC). The possible cancer-preventive activity of green tea constituents has been studied extensively, by us and others [141]. Many* in vitro* and* in vivo* studies have reported that consumption of green tea polyphenols (GTP) is associated with decreased risk and/or slower progression of PCa [142,143]. GTP inhibits prostate carcinogenesis by modulating one or more cell signaling pathways (NF-κB/MAPK/IGFR/COX-2), inhibiting many protein kinases, and suppressing the activation of transcription factors [144].

Among catechins, EGCG has been shown to be the most powerful with an anti-oxidant activity about 25–100 times more potent than that of vitamins C and E [145]. A vast amount of scientific literature is present showing the potential health benefits of EGCG attributable to green tea consumption. Various mechanisms have been proposed for the biological activities of EGCG such as anti-oxidant action, apoptosis induction, cell-cycle arrest, modulation of carcinogen-metabolizing enzymes, inhibition of mitotic signal transduction through modulation of growth factor receptor binding, and inhibition of DNA methylation [144]. In this context, recent studies showed that EGCG induced PCa cell death via downregulation of ID2 and up-regulation and stabilization of p53 [146]. EGCG promoted apoptosis associated with expression of CASP-9a splice variants in PCa cells, both alone and in combination with cisplatin [147,148]. EGCG provided protection against inflammation by suppressing proinflammatory cytokines, MMPs -2 and -9, independent of the AR expression and p53 status in PCa cells [149].

Green tea has higher concentrations of polyphenols, while black tea consumption has been shown to increase phenolic acids levels. Though clinical and pre-clinical studies provide evidence of green tea showing stronger chemopreventive effects as compared to black tea, concrete evidence from epidemiological studies is missing [150]. Additionally there still remain concerns about the bioavailability of EGCG and toxicity associated with its long-term use in clinical settings. Our group recently employed a nanochemoprevention approach involving chitosan-based nanoencapsulation of EGCG (Chit-nanoEGCG) for PCa cell growth inhibition, primarily addressing issues related to its bioavailability. Chit-nanoEGCG significantly inhibited tumor growth and suppressed PSA serum levels in athymic nude mice xenografted with 22Rν1 cells. In addition, there was significant induction of apoptosis and inhibition of tumor proliferation as evidenced by ADP-ribose polymerase cleavage, increase in BAX protein, decrease in Bcl-2, activation of CASPs, and reduction in proliferative markers Ki-67 and PCNA in the Chit-nanoEGCG treated groups as compared to control groups [151].

#### 3.4.3. Fisetin

Fisetin (FST; 3,7,3',4'-tetrahydroxyflavone) belongs to the flavonol subgroup of flavonoids, along with quercetin, myricetin and kaempferol. FST is primarily present in fruits and vegetables, such as strawberries, apples, persimmons, grapes, onions, and cucumbers [152]. Cell culture studies show that FST exerts anti-proliferative effect on human PCa cells. We have shown that FST selectively decreases the viability of LNCaP, 22Rν1, and PC3 cells with minimal effects on normal prostate epithelial cells [153,154]. FST induces apoptosis, cell cycle arrest, and inhibits androgen signaling and tumor growth in PCa models both* in vitro* and* in vivo*. FST suppressed cell proliferation by hypophosphorylation of eukaryotic translation initiation factor 4E-binding protein-1 and induced autophagic cell death in PCa cells through suppression of mTORC1 and mTORC2 complexes. In addition, FST acts as a dual inhibitor of mTORC1/C2. FST also activated the mTOR repressor TSC2 (tuberous sclerosis 2), commonly associated with inhibition of Akt and activation of AMPK [155].

TRAIL plays an important role in the defense against tumor cells. FST sensitizes androgen dependent LNCaP and androgen independent DU145 and PC3 cells to TRAIL-induced death. In addition, FST augmented TRAIL-mediated cytotoxicity and apoptosis in LNCaP cells by activating the extrinsic receptor-mediated and intrinsic mitochondrial apoptotic pathways. FST increased the expression of TRAIL-R1 and decreased NFκB activity [156]. Recently, we showed that FST inhibits YB-1, an important transcription factor that promotes epithelial-to-mesenchymal transition (EMT) in PCa. YB1 is overexpressed in PCa and has a functionally inverse relationship with e-cadherin, which is a marker for EMT. During PCa, endogenous EMT occurs which leads to induction of YB-1, which induces a mesenchymal phenotype both* in vitro* and* in vivo*. FST binds to the cold shock domain of YB-1 protein as shown by *in silico* docking studies and surfaces as an inhibitor of YB1 phosphorylation and MTA-1 expression. FST also inhibits EGF and TGF-β induced YB-1 phosphorylation and EMT in PCa cells [157]. Collectively, all these studies provide ample evidence that FST could be developed as an effective agent against PCa.

#### 3.4.4. Proanthocyanidins

Proanthocyanidins (PAC), commonly known as condensed tannins, are found abundantly in various plants and foods and contribute to organoleptic properties such as bitterness and astringency [158]. Food and nutritional supplements rich in PAC are known to have benefits in health promotion. PAC is primarily enriched in apple peel, red kidney beans, pinto beans, cacao beans, cocoa, grape seeds, blueberries, several nuts (peanuts, hazelnuts, *etc.*), sorghum , and cinnamon [159]. Cellular mechanisms involved in regulation of human PCa cells via blueberry fractions have been previously reported. MMPs are major mediators of extracellular matrix degradation and play an important role in PCa metastasis [160]. PAC, one of the primary flavonoids present in the blueberry fraction along with anthocyanin, caused down-regulation of MMP activity and up-regulation of endogenous tissue inhibitors of MMP’s (TIMP) activity in DU145 cells. The authors also reported the possible involvement of protein kinase C (PKC) and MAPK-associated events with a PAC-mediated decrease of MMP-2, -9 and increase in TIMP-1, -2 [161].

Another study examined the inhibitory effects of PAC isolates from wild and cultivated blueberries on proliferation of androgen dependent LNCaP and androgen independent DU145 cells. Differences in cell growth inhibition profile of LNCaP and DU145 cell lines indicated that PAC primarily affects the growth of androgen-dependent growth of PCa cells [162]. PAC isolated from cranberries via column chromatography was tested on DU145 tumor implants in athymic nude mice. PAC showed significant reduction in growth of the tumors explant cells* in vitro* and induced a complete regression in DU145 tumor implants* in vivo* [163]. Studies on PAC isolates from grape seeds demonstrated similar inhibitory effects on human PCa cells. Anti-proliferative and pro-apoptotic effects on LNCaP cells were primarily associated with decreased expression of androgen receptors. PAC mediated inhibition of CDKs, cyclins, and activation of tumor suppressors p21 and p27 was observed in both LNCaP and PC3 cells, along with changes in the Bcl-2/BAX ratio which favors apoptosis. PAC also induced cellular differentiation by increasing MAPK p44/42 [164].

In androgen-independent PCa, urokinase plasminogen activator (uPA) is implicated in cell migration and cancer metastasis. Treatments of PC3 cells with PAC-rich grape seed extract (PAC-GSE) have shown to regulate uPA expression and cell migration in a dose-dependent manner. Additional* in vitro* studies showed that PAC-GSE repressed DNA-binding activity of NFκB which in turn decreased NFκB-dependent uPA transcription [165]. Additional* in vitro* studies have found that PAC is an inhibitor of apoptosis suppressor proteins, NFκB, PI3K/Akt pathway, cytokines, angiogenesis factors, and many other molecular targets, which may contribute to anti-proliferative and pro-apoptotic effects in PCa and many other cancers [166]. Recently, a cancer prevention study II (CPS-II) was reported showing PAC intake and its inverse relationship of PCa risk in a cohort of US men, suggesting its potential efficacy against this deadly disease [167].

## 4. Limitations and Future Directions

Our understanding of the molecular aspects of PCa has progressed a lot in the recent years, but the overall PCa incidence and mortality remains a significant concern. Taking all the present findings to date into consideration, further research in PCa treatment and prevention remains critically important to target this deadly disease. A major limitation for the effectiveness of polyphenols in disease prevention is their bioavailability. It differs greatly among various polyphenols and the most abundant ones in our diet are not necessarily those having the best bioavailability profile. The identification and quantification of polyphenol metabolites with a focus on their potential biological activity should be the emphasis in future. To improve the bioavailability, dedicated strategies need to be implemented and it is necessary to determine whether these strategies actually translate into increased biological activity. Suitable animal models and appropriate doses should be used to demonstrate the true health benefits of dietary polyphenols before clinical trials in humans are initiated.

With current advancements in available technologies, it is now possible to interpret specific molecular events responsible for the anti-tumor effects of each individual polyphenol. This could allow present and future investigators to design preclinical studies to establish the scientific basis upon which more human studies can be planned. This will potentially help eliminate any existing disagreements with regards to past epidemiological and clinical studies. Motivated research on polyphenols and its anti-tumor effects will identify new molecules that can be studied and used for PCa prevention and treatment, both alone and in combination with existing therapies. Despite the various health benefits, polyphenols need to undergo similar analyses used for development of new therapeutic drugs. The results from such approaches shall determine the pharmacokinetics profiles of the compounds, as well as confirm the presumed interactions with other molecules. Several polyphenols possess synergistic characteristics with cancer chemotherapeutic agents. Hence, an appropriate combination of polyphenols with existing chemotherapeutics will lead to a reduction in side effects without decreasing the chemotherapeutic effects. Furthermore, dietary polyphenols are promising molecules for chemoprevention of PCa as they are safe and inexpensive, especially in patients at increased risk of PCa due to their genetic background or long-term exposure to carcinogens.

## 5. Conclusions

Studies in literature provide ample evidence that polyphenols have the potential to prevent PCa risk. Patients diagnosed with PCa have depleted antioxidant levels in blood [168] and increased levels of lipid peroxidation [169,170]. Dietary polyphenols in plasma have been shown to influence PCa risk by regulating inflammatory genes and repairing oxidative DNA damage [171,172]. In addition, there are also interactions between different dietary polyphenols, which could modify PCa risk through both anti-oxidant and non-anti-oxidant mechanisms [173]. The effect of dietary polyphenols on PCa remains inconclusive until well designed clinical trials are initiated to prove their efficacy in humans. The development of PCa is driven mainly by signaling pathways; hence, multi-targeted therapy approach should be employed to evade and avoid drug resistance. Further precise studies are needed to find the specific target of each polyphenol so that a combination regimen could be developed. Thus, the association of dietary polyphenols and their influence on PCa risk in target populations and patients renders a very promising tool for prevention and treatment of PCa.
